# Garlic bioactive substances and their therapeutic applications for improving human health: a comprehensive review

**DOI:** 10.3389/fimmu.2024.1277074

**Published:** 2024-06-10

**Authors:** Mohamed T. El-Saadony, Ahmed M. Saad, Sameh A. Korma, Heba M. Salem, Taia A. Abd El-Mageed, Samar Sami Alkafaas, Mohamed I. Elsalahaty, Sara Samy Elkafas, Walid F. A. Mosa, Ahmed Ezzat Ahmed, Betty T. Mathew, Noor A. Albastaki, Aysha A. Alkuwaiti, Marawan K. El-Tarabily, Synan F. AbuQamar, Khaled A. El-Tarabily, Salam A. Ibrahim

**Affiliations:** ^1^Department of Agricultural Microbiology, Faculty of Agriculture, Zagazig University, Zagazig, Egypt; ^2^Department of Biochemistry, Faculty of Agriculture, Zagazig University, Zagazig, Egypt; ^3^Department of Food Science, Faculty of Agriculture, Zagazig University, Zagazig, Egypt; ^4^School of Food Science and Engineering, South China University of Technology, Guangzhou, China; ^5^Department of Poultry Diseases, Faculty of Veterinary Medicine, Cairo University, Giza, Egypt; ^6^Department of Soils and Water, Faculty of Agriculture, Fayoum University, Fayoum, Egypt; ^7^Molecular Cell Biology Unit, Division of Biochemistry, Department of Chemistry, Faculty of Science, Tanta University, Tanta, Egypt; ^8^Biochemistry Division, Department of Chemistry, Faculty of Science, Tanta University, Tanta, Egypt; ^9^Production Engineering and Mechanical Design Department, Faculty of Engineering, Menofia University, Menofia, Egypt; ^10^Faculty of Control System and Robotics, Information Technologies, Mechanics and Optics (ITMO) University, Saint-Petersburg, Russia; ^11^Plant Production Department (Horticulture-Pomology), Faculty of Agriculture, Saba Basha, Alexandria University, Alexandria, Egypt; ^12^Biology Department, College of Science, King Khalid University, Abha, Saudi Arabia; ^13^Department of Biology, College of Science, United Arab Emirates University, Al Ain, United Arab Emirates; ^14^Department of Chemistry, College of Science, United Arab Emirates University, Al Ain, United Arab Emirates; ^15^Faculty of Medicine, University of Debrecen, Debrecen, Hungary; ^16^Harry Butler Institute, Murdoch University, Perth, WA, Australia; ^17^Food Microbiology and Biotechnology Laboratory, Food and Nutritional Science Program, North Carolina A&T State University, Greensboro, NC, United States

**Keywords:** *Allium sativum*, bioactive compounds, functional foods, human diseases, human health, mechanisms of action, toxicity

## Abstract

Garlic (*Allium sativum* L.) is a widely abundant spice, known for its aroma and pungent flavor. It contains several bioactive compounds and offers a wide range of health benefits to humans, including those pertaining to nutrition, physiology, and medicine. Therefore, garlic is considered as one of the most effective disease-preventive diets. Many *in vitro* and *in vivo* studies have reported the sulfur-containing compounds, allicin and ajoene, for their effective anticancer, anti-diabetic, anti-inflammatory, antioxidant, antimicrobial, immune-boosting, and cardioprotective properties. As a rich natural source of bioactive compounds, including polysaccharides, saponins, tannins, linalool, geraniol, phellandrene, β-phellandrene, ajoene, alliin, S-allyl-mercapto cysteine, and β-phellandrene, garlic has many therapeutic applications and may play a role in drug development against various human diseases. In the current review, garlic and its major bioactive components along with their biological function and mechanisms of action for their role in disease prevention and therapy are discussed.

## 1 Introduction

Garlic (*Allium sativum* L.) is a member of the Liliaceae family (1). The genus *Allium* contain hundreds of species, including garlic, onion, shallot, leek and chive, that are known for their physiologically active secondary metabolites (2). Garlic is an ancient crop that is native to Central and South Asia, and northeastern Iran (3). Garlic has compact bulbs, and each bulb consists of several bulblets/cloves that are covered with a white skin (4). Its products, including garlic oil macerates, essential oils (EOs), garlic powder, and aged garlic extract (5) exhibit a noteworthy therapeutic impact (6). Thus, they are economically important due to their nutraceutical characteristics and health advantages (7).

The raw garlic-derived compounds contain organosulfur components susceptible to oxidation, volatilization, and deterioration when exposed to unfavorable conditions such as light, oxygen, and high temperatures (8). These components are also thermodynamically unstable. Encapsulation methods utilizing a range of wall materials and technologies were implemented to enhance the stability and suitability of the bioactive constituents in garlic for use in the food industry. The principal organosulfur components found in garlic essential oil are as follows: diallyl sulfide (DAS), dimethyl trisulfide, diallyl tetrasulfide, methyl disulfide, allyl methyl trisulfide, diallyl trisulfide, allyl disulfide, and allyl methyl tetrasulfide (9). Aqueous and alcoholic garlic extract (GE) contain S-allyl-mercapto cysteine (SAMC), S-methyl-l-cysteine, S-propenyl-l-cysteine, and S-allyl-cysteine, all of which are derived from γ-glutamyl-S-allyl-L-cysteines (10).

This comprehensive review provides recent experimental and clinical reports. It will encompass an analysis of the safety profile, metabolism pathway, bioavailability, biological and therapeutic effects, food-related applicability, adulteration detection methods, potential toxicities, and bioactive compounds derived from garlic. We believe that by publishing this review paper, more people will be interested in garlic and that it will provide up-to-date scientific data for enhanced garlic use in human health as well as illness management.

## 2 Methodology

### 2.1 Study design

The study reviews relevant and current literature on bioavailability, biological and therapeutic effects, food-related applicability, adulteration detection methods, potential toxicities, and bioactive compounds derived from garlic.

### 2.2 Data collection

Data were collected from related past and recent textbooks, proceedings, and research articles using PubMed, Scopus, Google Scholar, and Web of Science. The keywords were *Allium sativum*, bioactive compounds, functional foods, human health, human diseases, mechanisms of action, and toxicity.

All kinds of related articles and abstracts were included. Criteria were set to studies published in the last five years as much as possible. Following the outlined searches, articles were chosen based on their relevance to the objective of this review. Articles providing information in clear relation to garlic active compounds and their pharmacological effects, with a clear indication of their action mechanisms, were also included. A total of 235 articles were included in this review.

## 3 Botanical description

Garlic is an herbaceous plant with a fragrant stem segregated into 6 to 12 bulblets named garlic cloves, linked by a fine shell to create the garlic head (11). The roots of garlic emerge from the disc’s basal part and can grow to a depth of at least 80 cm closer to the plant’s base. Its leaves are morphologically long, narrow, and flat; however, the tip is cylindrical and sharp (12). The flowers are tiny and whitish purple.

Garlic cultivation necessitates thick clay soil, humus, and enough water (13). A 70–80 cm height characterizes the garlic plant; several leaves are 13–15, the weight of the bulb is 50 g with a diameter of 5 cm, the number of teeth is 12–13, the period of growth in sowing winter is 250 days, and 40-day delay (14). **Table 1** summarizes the different parts of garlic, its active compounds, and their effects.

**Table 1:** The active compounds and their biological activities associated with different garlic parts.

Active part	Active compounds	Molecular Formula and weight		Activity	References
Sulfur compounds					
Bulb	Methiin	C_4_H_9_NO_3_S	151.19	Anticancer; Antioxidant	(15)
Bulb	Ethiin	C_5_H_11_NO_3_S	165.21	Anticancer; Anti-inflammatory; Antimicrobial; Antioxidant	(15)
Bulb	Isoalliin	C_6_H_11_NO_3_S	177.22	Anticancer; Antimicrobial; Antioxidant	(15)
Leaf	Alliin	C_6_H_11_NO_3_S	177.22	Anti-inflammatory; Antimicrobial; Antioxidant	(16)
Bulb	2-Propenesulfenic acid	C_3_H_6_OS	90.14	Cardioprotective; Immunomodulatory	(17)
Bulb	Diallyl sulfide	C_6_H_10_S	114.21	Cardioprotective; Immunomodulatory	(18, 19)
Bulb	Dimethyl disulfide	C_2_H_6_S_2_	94.20	Antithrombotic	(19)
Bulb	2-Propenesulfinic acid	C_3_H_6_O_2_S	106.14	Cardioprotective; Immunomodulatory	(17)
Bulb	Methyl-(E-*Z*)-1-propenyl disulfide	C_4_H_8_S_2_	120.24	Antithrombotic; Cardioprotective; Immunomodulatory	(19)
Bulb	(*E-Z*)-1-Propenyl-2-propenyl disulfide	C_6_H_10_S_2_	146.30	Antithrombotic; Cardioprotective; Immunomodulatory	(19)
Bulb	(E-*Z*)-Methyl-1-propenyl disulfide-5-Oxide	C_4_H_8_OS_2_	136.24	Antithrombotic; Cardioprotective; Immunomodulatory	(17)
Bulb	2-Propeneperthiol	C_3_H_6_S_2_	106.21	Antimicrobial; Antioxidant	(20)
Bulb	Allyl methyl disulfide	C_4_H_8_S_2_	120.24	Antimicrobial; Antioxidant	(19)
Bulb	Allyl methyl thiosulfinates	C_4_H_8_OS_2_	136.24	Antimicrobial; Antioxidant	(20)
Bulb	Isoallyl methyl thiosulfinates	C_4_H_8_OS_2_	136.24	Antimicrobial; Antioxidant	(20)
Bulb	*S*-Allylmercapto-L-cysteine	C_6_H_11_NO_2_S_2_	193.30	Antimicrobial; Antioxidant	(21)
Bulb	*γ*-L-Glutamyl-*S*-allylthio-L-cysteine	C_11_H_18_N_2_O_5_S_2_	322.40	Cardioprotective; Immunomodulatory	(22)
Bulb	Allithiamine	C_15_H_22_N_4_O_2_S_2_	354.50	Anticancer; Anti-inflammatory; Antimicrobial; Antioxidant	(23)
Bulb	Methyl methanethiosulfinate	C_2_H_6_OS_2_	110.20	Anticancer; Anti-inflammatory; Antimicrobial; Antioxidant	(20)
Bulb	Dihydroasparagusic acid	C_4_H_8_O_2_S_2_	152.20	Anticancer; Anti-inflammatory; Antimicrobial; Antioxidant	(24)
Bulb	Dimethyl trisulfide	C_2_H_6_S_3_	126.30	Anticancer; Anti-inflammatory; Antimicrobial; Antioxidant	(19)
Bulb	Allyl-1-propenyl trisulfide	C_6_H_10_S_3_	178.30	Anticancer; Anti-inflammatory; Antimicrobial; Antioxidant	(25)
Bulb	Methyl-2-propenyl trisulfide	C_4_H_8_S_3_	152.30	Cardioprotective; Immunomodulatory	(26)
Bulb	Diallyl trisulfide	C_6_H_10_S_3_	178.30	Antithrombotic; Cardioprotective; Immunomodulatory	(25)
Bulb	Ajoene	C_9_H_14_OS_3_	234.40	Anticancer; Anti-inflammatory; Antimicrobial	(27)
Bulb	Allyl propyl trisulfide	C_6_H_12_S_3_	180.40	Antimicrobial; Antioxidant	(19)
Bulb	Ajocysteine	C_9_H_15_NO_3_S_3_	281.40	Antimicrobial; Antioxidant	(27)
Bulb	1,2-Dithiolene	C_3_H_4_S_2_	104.20	Antimicrobial; Antioxidant	(19)
Leaf	Foliogarlic trisulfane	C_12_H_16_O_6_S_3_	352.45	Antimicrobial; Antioxidant	(28)
Leaf	Foliogarlic disulfanes	C_12_H_16_O_6_S_2_	320.38	Antimicrobial; Antioxidant	(28)
Bulb	Garlicnin	C_9_H_16_O_2_S_2_	220.35	Antimicrobial; Antioxidant	(29)
Bulb	1,2-Dithiolane	C_3_H_6_S_2_	106.21	Antimicrobial; Antioxidant	(19)
Amino acids and their derivatives					
Bulb	Valine	C_5_H_11_NO_2_	117.15	Antimicrobial; Antioxidant	(30)
Bulb	α-Aminobutyric acid	C_4_H_9_NO_2_	103.12	Antimicrobial; Antioxidant	(31)
Bulb	Choline	C5H_14_NO^+^	104.17	Antimicrobial; Antioxidant	(30)
Bulb	Proline	C_5_H_9_NO_2_	115.13	Antimicrobial; Antioxidant	(30)
Bulb	5-Oxoproline	C_5_H_7_NO_3_	129.11	Antimicrobial; Antioxidant	(30)
Bulb	Aspartic acid	C_4_H_7_NO_4_	133.10	Antimicrobial; Antioxidant	(30)
Bulb	Glutamic acid	C_5_H_9_NO_4_	147.13	Antimicrobial; Antioxidant	(30)
Bulb	Lysine	C_6_H_14_N_2_O_2_	146.19	Antimicrobial; Antioxidant	(30)
Bulb	Hydroxylysine	C_6_H_14_N_2_O_3_	162.19	Antimicrobial; Antioxidant	(30)
Bulb	Arginine	C_6_H_14_N_4_O_2_	174.20	Antimicrobial; Antioxidant	(30)
Bulb	Leucine	C_6_H_13_NO_2_	131.17	Antimicrobial; Antioxidant	(30)
Bulb	Se-Methylselenocysteine	C_4_H_9_NO_2_Se	182.09	Cardioprotective	(28)
Bulb	γ-Glutamyl-Se-methylselenocysteine	C_9_H_15_N_2_O_5_Se_-_	310.20	Immunomodulatory	(28)
Oxygenic compounds					
Bulb	Myristic acid	C_14_H_28_O_2_	228.37	Cardioprotective; Immunomodulatory	(30)
Bulb	Palmitic acid	C_16_H_32_O_2_	256.42	Antimicrobial; Antioxidant	(32)
Bulb	Oleic acid	C_18_H_34_O_2_	282.50	Antimicrobial; Antioxidant	(30)
Bulb	Aminoacrylic acid	C_3_H_5_NO_2_	87.08	Antimicrobial; Antioxidant	(30)
Bulb	Lactic acid	C_3_H_6_O_3_	90.08	Anticancer; Anti-inflammatory; Antimicrobial; Antioxidant	(30)
Bulb	Succinic acid	C_4_H_6_O_4_	118.09	Anticancer; Anti-inflammatory; Antimicrobial; Antioxidant	(30)
Bulb	Malic acid	C_4_H_6_O_5_	134.09	Anticancer; Anti-inflammatory; Antimicrobial; Antioxidant	(30)
Bulb	Arabinoic acids	C_5_H_10_O_6_	166.13	Anticancer; Anti-inflammatory; Antimicrobial; Antioxidant	(30)
Bulb	Mandelic acid	C_8_H_8_O_3_	152.15	Anticancer; Anti-inflammatory; Antimicrobial; Antioxidant	(30)
Bulb	Carvacrol	C_10_H_14_O	150.22	Antimicrobial; Antioxidant	(30)
Bulb	Carvone	C_10_H_14_O	150.22	Antimicrobial; Antioxidant	(30)
Bulb	*p*-Tert-butylphenol	C_10_H_14_O	150.22	Antimicrobial; Antioxidant	(32)
Bulb	Linalool	C_10_H_18_O	154.25	Antimicrobial; Antioxidant	(30)
Bulb	4-(1,1-Dimethylpropyl) phenol	C_11_H_16_O	164.24	Antimicrobial; Antioxidant	(32)
Bulb	2-Methylene-4-pentenal	C_6_H_8_O	96.13	Antimicrobial; Antioxidant	(19)
Bulb	Ammonium pyruvate	C_3_H_7_NO_3_	105.09	Anticancer; Anti-inflammatory; Antimicrobial; Antioxidant	(33)
Bulb	Geraniol	C_10_H_18_O	154.25	Anticancer; Anti-inflammatory; Antimicrobial; Antioxidant	(26)
Bulb	2-Propen-1-ol	C_3_H_6_O	58.08	Anticancer; Anti-inflammatory; Antimicrobial; Antioxidant	(32)
Bulb	2-Hydroxy propanamide	C_3_H_7_NO_2_	89.09	Anticancer; Anti-inflammatory; Antimicrobial; Antioxidant	(32)
Bulb	Retinoyl-β-glucuronic acid	C_26_H_36_O_8_	476.60	Antimicrobial; Antioxidant	(30)
Phenolic compounds					
Bulb	Eugenol-1-O-rutinoside	C_22_H_32_O_11_	472.48	Cardioprotective	(34)
Root	*N*-Feruloyltyramine	C_18_H_19_NO_4_	313.30	Immunomodulatory	(35)
Root	*N*-Feruloyltyrosine	C_19_H_19_NO_6_	357.40	Anticancer; Anti-inflammatory	(36)
Husk waste	*p*-Coumaric acid	C_9_H_8_O_3_	164.16	Anticancer; Anti-inflammatory	(36)
Husk waste	Ferulic acid	C_10_H_10_O_4_	194.18	Anticancer; Anti-inflammatory	(36)
Husk waste	Caffeic acid	C_9_H_8_O_4_	180.16	Anticancer; Anti-inflammatory	(36)
Bulb	Apigenin	C_15_H_10_O_5_	270.24	Anticancer; Anti-inflammatory	(26)
Bulb	Quercetin	C_15_H_10_O_7_	302.23	Anticancer; Anti-inflammatory	(26)
Bulb	Myricetin	C_15_H_10_O_8_	318.23	Anticancer; Anti-inflammatory	(35)
Bulb	Naringenin	C_15_H_12_O_5_	272.25	Antimicrobial; Antioxidant	(37)
Bulb	Catechin	C_15_H_14_O_6_	290.27	Antimicrobial; Antioxidant	(30)
Bulb	Gallocatechol	C_15_H_14_O_7_	306.27	Antimicrobial; Antioxidant	(30)
Bulb	Tangeretin	C_20_H_20_O_7_	372.40	Antimicrobial; Antioxidant	(37)
Bulb	Nobiletin	C_21_H_22_O_8_	402.40	Antimicrobial; Antioxidant	(37)
Bulb	Rutin	C_27_H_30_O_16_	610.50	Anticancer; Anti-inflammatory; Antimicrobial; Antioxidant	(26)
Bulb	Theophylline	C_7_H_8_N_4_O_2_	180.16	Antimicrobial; Antioxidant; Anticancer Anti-inflammatory	(30)
Bulb	Nicotine	C_10_H_14_N_2_	162.23	Antimicrobial; Antioxidant; Anticancer Anti-inflammatory	(30)
Bulb	Cyanidin	C_15_H_11_O_6_	287.24	Antimicrobial; Antioxidant; Anticancer Anti-inflammatory	(30)
Bulb	Delphinidin	C_15_H_11_ClO_7_	338.69	Antimicrobial; Antioxidant; Anticancer; Anti-inflammatory	(30)
Bulb	Anthocyanin	C_15_H_11_O	207.25	Antimicrobial; Antioxidant; Anticancer; Anti-inflammatory	(30)
Bulb	Pelargonidin	C_15_H_11_O	271.24	Antimicrobial; Antioxidant; Anticancer; Anti-inflammatory	(30)

## 4 Phytochemical compounds in garlic

Approximately 184 diverse types of active secondary metabolites are found in garlic (38). Almost 90% of these metabolites are characterized as the following: 70 organosulfur, 29 saponins, 16 flavonoids, 12 amino acids, and phenyl prostanoids 10 for each of phenyl prostanoids, 7 alkaloids, in addition to 3 compounds of fatty acids, and selenium derivatives (38). In leaves, the active secondary metabolites represent nine compounds, while in roots and husk wastes, there are 6 and 3 compounds, respectively. In general, there are 166 plant chemicals in bulbs and cloves (39).

### 4.1 Bioactive compounds of garlic

Due to its many phytochemicals, minerals, and dietary fiber, garlic is considered a valuable spice. Moderate amounts of selenium, calcium, magnesium, manganese, iron, vitamins A, C, and B complex, and sodium are also present. Potassium, zinc, sulfur, and phosphorus are all found in substantial concentrations (40). Dietary fiber comprises approximately 1.5% of the garlic bulb’s composition, followed by protein (2%), organosulfur substances (2.3%), carbohydrates (28%), and water (65%) (41).

**Figure 1:**
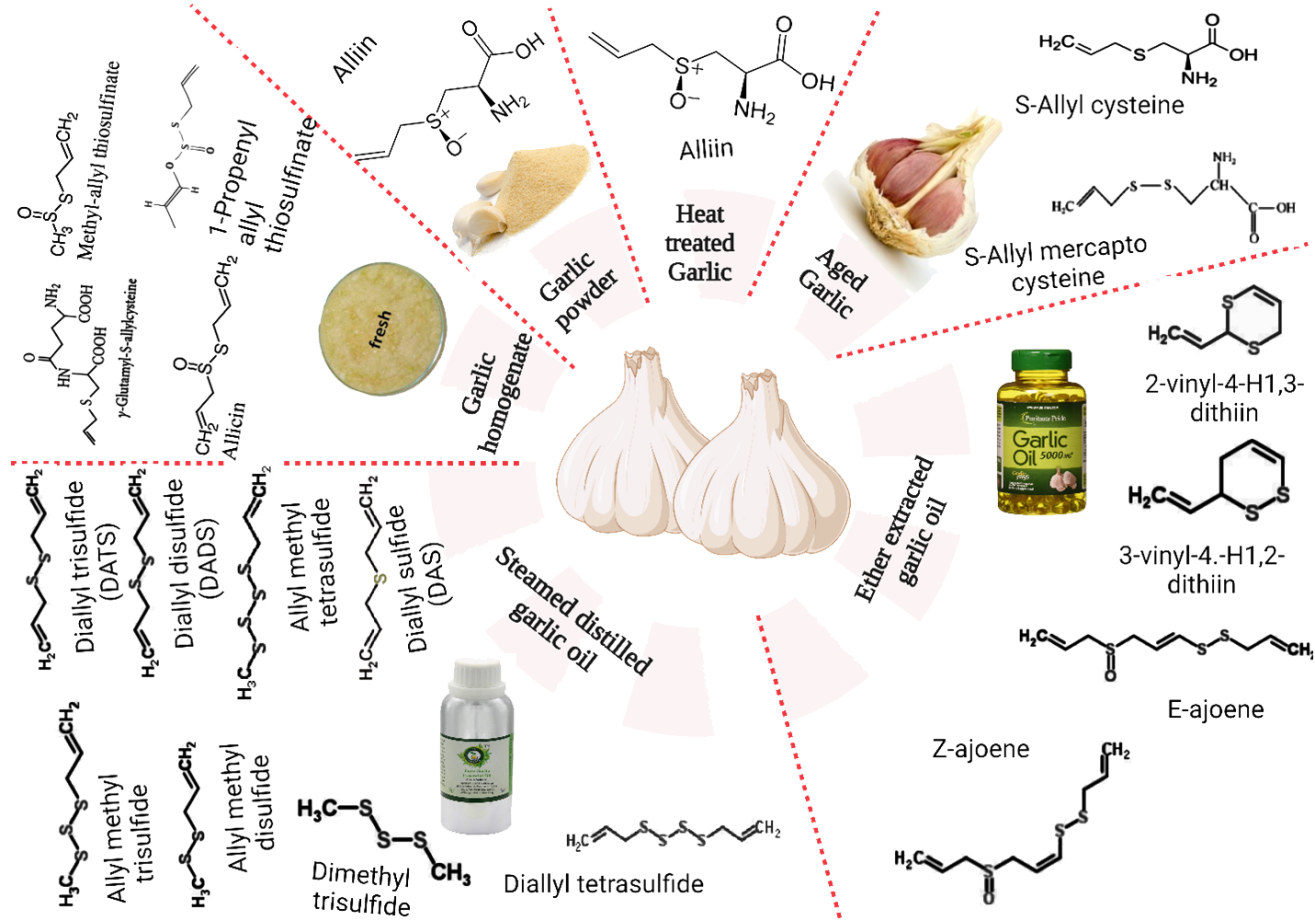
Major water and oil-soluble sulfur compounds found in garlic.

The principal bioactive components of these substances have recently sparked widespread scientific attention. These include a variety of substances such as phenols, enzymes (myrosinase, allinase, peroxidase), polysaccharides, saponins, tannins, linalool, geraniol, phellandrene, β-phellandrene, ajoene, alliin, SAMC, and β-phellandrene. A limited selection of the recognized polyphenolic constituents found in the matrix of garlic include apigenine, kaempferol-3-O-glucoside-7-O-alpha-l-rhamnoside, kaempferol 3,7-di-O-rhamnoside, luteoline, and kaempferol-3-O-glucoside (42). Furthermore, it comprises 17 amino acids, of which 8 are primary (43). Although intact garlic typically comprises advantageous constituents, further analysis has revealed the presence of additional substances, including DAS, dithiine, diallyl disulfide (DADS), allicin, and ajoene, which are the result of diverse chemical reactions occurring during pulverizing or chopping (44). **Figure 1** depicts the major water-soluble and oil-soluble sulfur compounds present in garlic.

The primary chemical composition and profiles of bioactive components in garlic are significantly influenced by various factors, such as genotype, irrigation, temperature, light, and fertilization during the pre-harvest period; harvest and post-harvest conditions, ventilation, relative humidity, and temperature (45). As illustrated in **Figure 1**, dehydrated or whole garlic contains organosulfur components such as γ-glutamylcysteine and alliin (S-allylcysteine sulfoxide). These components are biogenic facilitators for generating additional sulfur byproducts throughout garlic production and heating (46). Alliinase enzymes (EC 4.4.1.14), which are initially limited to the cells of the vascular bundle sheath and rapidly convert alliin (which is categorized in mesophyll cells) to allicin, are released during cutting, crushing, or timidity (47).

### 4.2 Allicin contents

Allicin, the principal S-molecule in garlic, comprises approximately 70–80% of its bioactive compounds and is accountable for imparting its distinctive aroma (48). Allicin rapidly decomposes and generates other stable organosulfur constituents, including DADS, diallyl trisulfides, vinyldithiines (3-vinyl-4H-1,2-dithiin and 2-vinyl-4H-1,3-dithiin), DAS, and ajoenes (49). These constituents are sensitive to organic solvents, oxygen, temperature, and pH. The various physiological and biological attributes of garlic, including its antidiabetic, anti-cancer, anti-inflammatory, antiobesity, antioxidant, antifungal, antibacterial, and immunomodulatory properties, can be primarily attributed to the bioactive constituents present in its matrix. These include organosulfur compounds (e.g., allicin, DAS, allyl propyl disulfide, diallyl polysulfides, vinyldithiins, DADS, and diallyl trisulfide) (49).

### 4.3 E- and z-ajoene content

Ajoene is a group of organosulfur compounds with garlic-like characteristics (45). The biological activities of garlic are linked to various compounds, including ajoene, produced through allicin breakdown and non-enzymatic repair (50). In cancer cells, it has been shown triggering apoptosis (51); thus having an anti-cancer impact. Ajoene has been demonstrated to decrease the proliferation of lung cancer cells in some studies (52). Ajoene is also an antifungal medication that is safe and successful in treating chromoblastomycosis and dermatophytosis (53). Its inhibitory dose can be below 20 µg/ml (54, 55). Choi et al. (56) employed ajoene at 3 µg/ml extract concentrations to boost macrophage antimycobacterial activity. Ajoene can also disrupt biological machinery and is lethal to cancer cells at low µM concentrations (57).

## 5 Bioavailability of active compounds after metabolism

Allicin and other biological sulfur compounds found in garlic are believed to be the primary pharmacologically active components of the matrix due to their potent anticancer, antibacterial, and antioxidant properties. Absorption in the intestines is a critical factor in the health-promoting effects of the biological components in garlic. The absorption rate significantly influences the availability of these compounds (58).

Investigating how compounds are absorbed into the body, particularly in relation to food, is a fascinating trend in research across different fields (58). Based on *in vitro* experiments concerning the potential of raw garlic and its preparation in cancer treatment, *in vivo* studies and clinical trials have been initiated, albeit their contradictory results (59). Indeed, variations exist regarding the bioavailability of the sulfur-containing primary groups when comparing fresh garlic to specific culinary and medicinal preparations that incorporate garlic. *In vivo* research investigated the bioavailability of allicin from 13 garlic supplements and 9 meals in 13 participants by quantifying allyl methyl sulfide, the principal garlic metabolite, in their breath. The bioavailability of allicin supplements was found to be higher than freshly pulverized garlic (59).

Allicin is responsible for the majority of the pharmacological effects of raw garlic. When inhibiting enzymes in the gastrointestinal tract, it is swiftly biotransformed (half-life <1 min) to allyl mercaptan. Upon consuming a substantial quantity (25 g) of crushed fresh garlic, allicin and its metabolites have been detected in the urine, feces, and blood (60). Allicin is metabolized into secondary compounds, including E-ajoene, 2-ethenyl-4H-1, 3-dithiin, and DADS, much like how rapidly it exited the body following intravenous infusion (61).

Furthermore, approximately 30 min after ingestion, the bioavailability of allicin from enteric-coated tablet formulations varied between 36 and 104%. However, when inhaled with a protein-rich meal, the bioavailability decreased from 22 to 57% (59). The bioavailability of garlic capsules was 26–109% lower than that of non-enteric-coated tablets (59). Furthermore, the cysteine protein derivative interacts quantitatively with allicin at the temperature of the host gut to generate two equivalents of SAMC. This occurs when cysteine produced from ingested food protein interacts with allicin in garlic supplements in the gastrointestinal environment (59).

In an *in vivo* experiment using mice, the principal metabolites allyl methyl sulfone and allyl methyl sulfoxide were detected in the liver, stomach, plasma, and urine after DADS (200 mg/kg) was administered orally (62). Metabolic analysis of DADS radioactively tagged in mouse livers revealed that 70% of the radioactive substance was present in the cytoplasm; however, it did not exist in the form of DADS. DADS constituted 80% of the radioactivity (63). This research indicates that sulfur compounds derived from the metabolism of allicin are primarily responsible for physiological effects. One of the chemical compounds generated by these substances is hydrogen sulfide, which functions as a signal transduction substance within the human body (64).

Aged garlic extract is composed primarily of water-soluble organosulfur compounds, including S-allyl cysteine (SAC) and SAMC, which have a distinct pharmacokinetic profile compared to oil-soluble organosulfur elements (65). In addition, garlic is rapidly absorbed from the gastrointestinal tract, and SAC has a half-life of over 10 h and a renal clearance of over 30 h in humans. Based on SAC’s efficacy and safety assessment, it appears to significantly impact the biological characteristics (66). Effective *in vitro* extraction methods can reduce potential toxicity while enhancing the bioavailability of garlic. During the extraction of aged garlic extract, the garlic matrix’s undesirable constituents are transformed into S-components securely and consistently. Several toxicological investigations have also confirmed the safety of aged garlic extract (67).

It is noteworthy that although organic S-compounds (OSCs) have a low bioavailability in the host body, research indicates that incorporating garlic into one’s daily diet can provide sufficient quantities to support their biological effects. Aryl and methyl are the two most prevalent forms of OSCs identified in garlic extract (68). SAC was found to have a relatively high bioavailability in the blood plasma, kidneys, and liver of primary animal experiments involving canine and mouse models exposed to aqueous garlic extracts (69). Garlic yielded 570 μg of DADS, 2.5 mg of allicin, and 60 μg of SAC, all of which are equivalent to 1000 μg of diallyl trisulfides (69).

After ingestion, the purified allicin was not detected in the blood, urine, or feces (70). An element that contributes to the diminished biological accessibility of allicin is its tendency to form bonds with fatty acids and proteins across plasma membranes (71). Some hypotheses regarding these barriers encompassed the challenge of allicin evading the digestive tract via its interaction with the intestinal mucosa and its oxidative reaction with red blood cells (71). Furthermore, it was characterized by its high reactivity and rapid conversion to allyl mercaptan (AM) (71).

A simulated digestion method was employed to investigate the impact of temperature, duration, pH, and neutralization after acidification on thiosulfate (TS) synthesis from garlic powder and cloves (60). An optimal pH range of 4.5-5.0 was identified as the setting for synthesizing allicin, allyl 1-propenyl, and 1-propenyl allyl (72). The best formations of allyl methyl+methylallyl and 1-propenyl methyl+methyl 1-propenyl occurred between pH 6.5-7.0. Dimethyl generated thiosulfinate at pH 5.5 and no TS below pH 3.6 (72). However, the pH of the intestines and stomach did not significantly influence the bioavailability or gastrointestinal metabolism of allicin. Although overall temperature had a negligible impact on the production of TS, the allicin experienced the most excellent disintegration and degradation at 35°C (72). Hydrolyzing allicin and alliin produced water-soluble compounds with enhanced antioxidant, bioavailability, and stability properties (72).

It is noteworthy to mention that the abrasive sensation experienced during garlic consumption can be attributed to the vasodilatory effects of DADS and allicin, which are produced by allyl isothiocyanate neurons stimulated by capsaicin neural terminals (70). In addition, increasing the levels of bioavailable substances, including DAS, ajoene, diallyl trisulfide, DADS, and SAC, and altering the expression of the DMT1 mRNA gene, garlic consumption can enhance iron absorption from carbonyl iron (73). Shallots and leeks have also been proposed to increase iron bioavailability in cereals and legumes (74).

Evaluations of sulfur constituents in different garlic substrates via *in vitro* digestion revealed that garlic powder exhibited the most significant accessibility, followed by garlic cloves. Nevertheless, there was a reduction in allicin availability when it was ingested alongside high-protein meals due to the subsequent delayed gastric emptying (75). Diverse organic sulfides may be produced from garlic through its multifaceted processing. The organic sulfides in question demonstrate an extensive array of significant biological and therapeutic attributes (75). Additional research into the bioavailability of various organic sulfides is crucial for expanding the pharmaceutical and health product industries involving garlic.

## 6 Biological activities and nutraceutical properties of garlic

**Figure 2:**
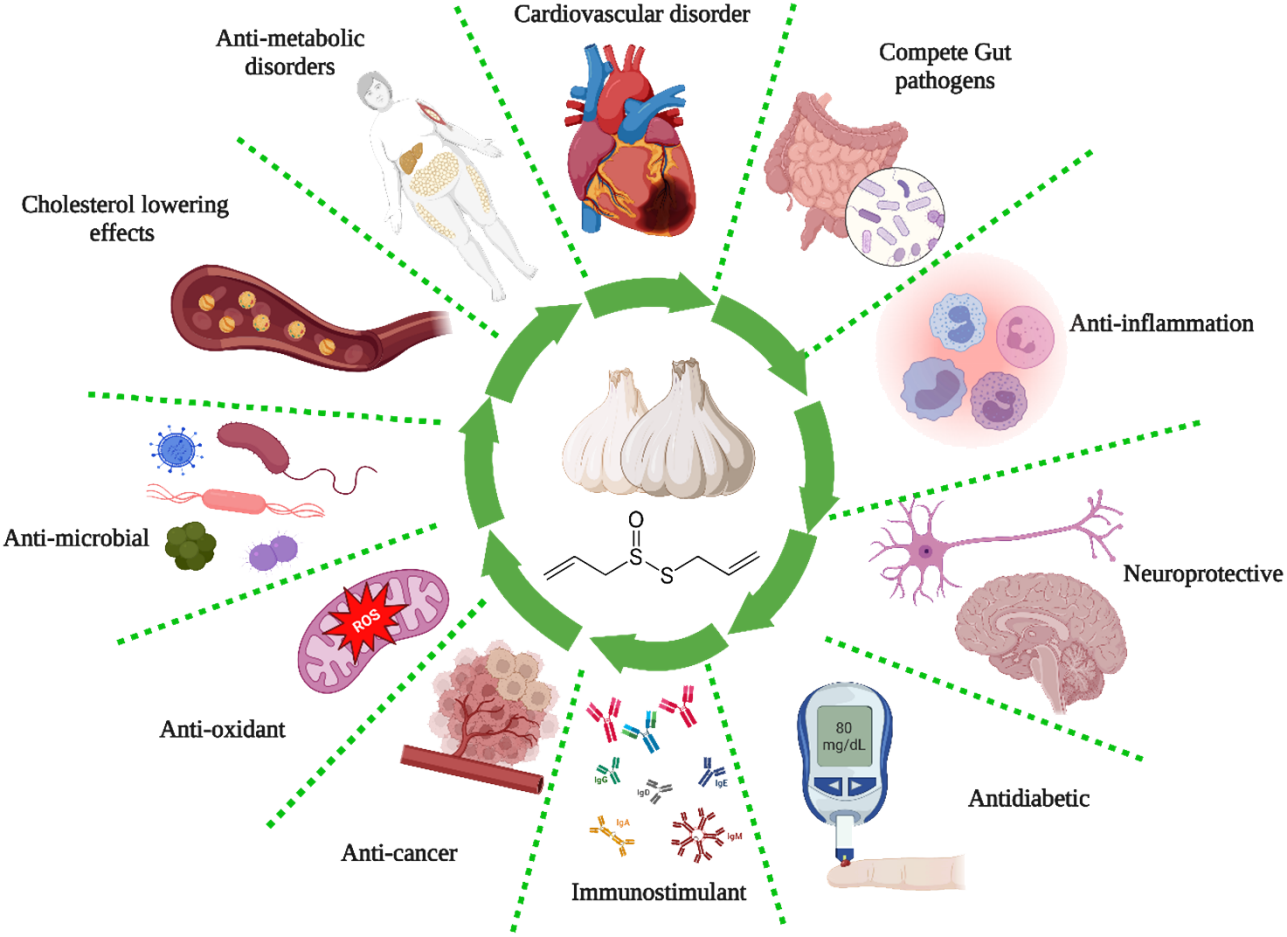
Biological activities of garlic.

The ancient Egyptians well-documented the antiquity of garlic as a remedy for respiratory and gastrointestinal ailments. They also incorporated it into a diverse array of nutritional and therapeutic products (76). Louis Pasteur was the initial proponent of the antibacterial properties of garlic in 1858. The enzymatic activity of alliin alkyl-sulfonate-lyase transforms the inactive constituent of garlic, alliin (a sulfoxide derivative of the cysteine amino acid), into allicin (diallyl thiosulfinate), which is the bioactive element accountable for the antibacterial properties of garlic (76). **Table 2** and **Figure 2** summarize the different biological activities of garlic and garlic products.

**Table 2:** Biological activities of garlic and garlic-derived compounds.

Extract/Product	Active compound	Activity	Host	Mechanism	References
**Aged garlic extract**	Eruboside B	*In vivo* antioxidant	Mouse myoblasts challenged by C2Cl2	Mitigating oxidative stress	(77)
	Iso- Eruboside B	*In vitro* antioxidant	Human endothelial cells	Scavenging H_2_O_2_, maintaining cell growth and protecting DNA from damage	(78)
	Group of active compounds	*In vitro* anticancer	SW480, SW620, A598, ECV304 cancer cells lines	Reducing invasive activity, reducing invasive activity, preventing cell division, and inhibiting endothelial cell tube formation	(79)
		*In vitro* anticancer	DLD-1 colon cancer cells	Inhibiting cell viability through inhibiting NF-κB activity	(80)
	Group of active compounds	*In vivo* anticancer	Adult albino rat challenged by cisplatin	Enhancing renal histological, ultrastructural, and biochemical alterations, including hemorrhage, glomerular atrophy, tubular necrosis, and degeneration	(81)
	Group of active compounds	*In vivo* anticancer	BALB/c mice injected with fibrosarcoma tumors	Enhancing the mice’s immunity to fibrosarcoma and inhibit tumor development.	(82)
	Ethyl linoleate	*In vitro* anti-inflammatory	RAW 264.7 macrophages induced by lipopolysaccharide	Limiting prostaglandin E-2 and NO release	(83)
	Allicin	*In vivo* anti-inflammatory	BALB/c mice	Preventing the inflammatory reaction	(84)
			40 Obese patients	Providing osteoarthritis relief	(85)
	14-KDa protein	*In vitro* anti-inflammatory	J774A1 macrophages induced by lipopolysaccharide	Impairing proinflammatory cytokines, including NO, TNF-α, and IL-1β	(86)
	Fru-Arg	*In vitro* neuroprotection	Microglial cells from murine BV-2 induced by lipopolysaccharide	Regulating protein expression in mitigating the oxidative stress causes, reducing nerve inflammation	(87)
	Group of active compounds	*In vivo* neuroprotection	Wister rats	Reducing the degradation of working memory	(88)
	Group of active compounds	*In vivo* protection of the digestive system		Preventing the ulceration caused by indomethacin	(89)
	Group of active compounds	*In vivo* protection of the digestive system	Albino rats	Restoring the damaged stomach mucosa caused by indomethacin, reducing the overall number of microbes in the stomach	(90)
	Allicin	*In vivo* protection of the digestive system	Induced colitis mice	Dextran sulfate sodium inducing ulcerative colitis, which can be alleviated by garlic extract	(91)
	Group of active compounds	Cohort study cardiovascular	41 patients with hypercholesterolemia	Reducing the activity of lipid hydroperoxide and serum myeloperoxidase, reducing F2-isoprostane concentrations in plasma and urine	(92)
	Group of active compounds	Cohort study immunity	56 healthy human participants	Reducing the incidence of cold and influenza Enhancing the immune system’s performance	(93)
	Allicin	*In vitro* antimicrobial	*Aspergillus versicolor* and *Penicillium expansum*	Inhibiting microbial population	(94)
	Garlic oil	*In vitro* antimicrobial	*Penicillum citrinum, Burkholderia cepacian*	Inhibiting microbial population	(95)
	Garlic oil	*In vitro* antimicrobial	*Penicillium funiculosum*	Inhibiting microbial population	(96)
	Garlic oil	*In vitro* antimicrobial	*Candida albicans*	Disrupting bacteria’s regular metabolic processes	(97)
	Garlic oil	*In vitro* antimicrobial	*Staphylococcus aureus, Escherichia* *coli* and *Bacillus subtilis*	Inhibiting microbial population	(98)
**Crude garlic extract**	Allicin	*In vitro* anticancer	Human gastric cancer cell lines	Inhibiting cell proliferation through increasing p53 activity and inhibiting Bcl-2 activity	(99)
	DATS	*In vitro* anticancer		Blocking cell cycle, decreasing cell viability, through increasing p53 activity and inhibiting Bcl-2 activity	(100)
		*In vitro* anticancer	Breast cancer cell	Decreasing cell viability through inhibiting angiogenesis	(101)
	SAC	*In vitro* anticancer	Epithelial ovarian cancer cell line	Inhibiting cell growth and inducing cell cycle arrest in the G1/S phase, increasing apoptosis, decreasing cell migration, decreasing pro-caspase-3, Parp-1, and Bcl-2 expression and boosting active caspase-3 and Bax, decreasing Wnt5a expression	(102)
	SAMC	*In vitro* anticancer	Human colorectal cancer cell line (SW620)	Reducing cell viability, cell growth, inducing apoptosis	(103)
		*In vitro* anticancer	Hep3B cancer cell line	Reducing the S phase and extending the G0/G1 phases	(104)
	SPRC	*In vitro* anticancer	Pancreatic cancer cells line	Reducing cell viability, cell growth, inducing apoptosis through inducing G2/M-phase and regulating JNK protein level	(105)
	Lipophilic compounds	*In vitro* anticancer	Hep-G2, Caco-2, PC-3, MCF-7, and TIB-71 cancer cell lines	Decreasing cancer cell viability by 90% on the third day of the experiment	(106)
	Ajoene	*In vitro* anticancer	Breast cancer cell	Reducing cell viability and cell growth, inducing apoptosis through folding proteins in cancer cell endoplasmic reticulum	(51)
	Alliin	*In vitro* anticancer	Gastric cancer cell lines	ROS production, mitochondrial membrane potential reduction through Bax/Bcl-2, and cytochrome C upregulation	(99)
**Garlic ethanolic extract**	Group of active compounds	*In vitro* anticancer	Bladder cancer cell	Reducing cell viability, cell growth, inducing apoptosis through Inducing G2/M-phase	(107)
	Group of active compounds	Cohort study anti-cancer	Patients treated with chemotherapy for hematologic cancers	Showing protective impact on febrile neutropenia in subgroups with reduced risk	(108)
	Group of active compounds	*In vivo* hepatoprotective	Wister rats	Enhancing plasma biochemical markers of liver function, including ALP, AST, urea, and creatinine	(109)
	SAC	*In vivo* neuroprotection	Wister rats	Mitigating oxidative stress, neuroinflammatory activity, astrogliosis, and acetylcholinesterase activity	(110)
	Ajoene	*In vivo* neuroprotection	Gerbils	Delaying I/R-induced neuronal death and gliosis in the hippocampus’s area through inhibiting lipid peroxidation	(111)
**Black garlic methanolic extract**	Group of active compounds	*In vivo* hepatoprotective	Rat hepatocytes	Interfering with oxidative stress, apoptosis, lipid peroxidation, and inflammation Alloxan-induced liver damage attenuation	(112)
		*In vivo* antiobesity	Male rats fed a high-fat diet	Decreasiing the weight and reduce the amount of fat in the retroperitoneal, epididymal, and mesenteric regions.	(113)
	Garlic oil	*In vivo* antiobesity		Controlling lipid metabolism, reducing weight	(114)
		*In vivo* antiobesity		Mitigating the effect of a high-fat diet on body mass and adipose tissue mass	(115)
	DADS	*In vivo* protection of the digestive system	Small intestine	Facilitating defecation by stimulating gastrointestinal peristalsis and promoting gastrointestinal emptying.	(116)
	DAS	*In vitro* protection of the digestive system	Stimulated intestinal cells	Reducing interferon-induced protein-10 and IL-6 and inhibiting NO and STAT-1 expression.	(117)
	DAS	*In vivo* protection of the digestive system	Male ICR mice	Enhancing colitis brought on by dinitro benzenesulfonic acid	(117)
**Raw garlic**	Group of active compounds	Cohort study Anti-cancer	17 volunteers	Seven genes are upregulated, including NFAM1, AHR, HIF1A, JUN, ARNT, OSM, and REL.	(118)
	Group of active compounds	*In vivo* antidiabetic	Diabetic rats	Preventing diabetic retinopathy, blood glucose, enhancing weight, and retinal tissue morphological alterations	(119)
	Group of active compounds	Cohort study Antidiabetic	768 type 2 diabetes patients	Producing glycosylated hemoglobin and Fructosamine reduction	(120)
	Group of active compounds	Cohort study Digestive protection and antimicrobial activity	*H. pylori* infected 15 patients	Inhibiting urease activity in bacterial cells, reducing the *Helicobacter pylori* count in the stomach	(121)
	Allyl methyl sulfide	*In vivo* cardiovascular	Diabetic rats	Maintaining cardiac function by inducing the pathway of sirtuin 3-manganese superoxide dismutase.	(122)
	Allyl methyl sulfoxide	*In vivo* cardiovascular	Sprague–Dawley rats	Reducing the remodeling of myocardial hypertrophy produced by isoproterenol	(123)
	Group of active compounds	Cohort study cardiovascular	30 diabetic patients	Reducing the content of cholesterol LDL and increase the HDL	(124)
	Essential oils	*In vivo* immunity	Wister rats	Several immunological indices of rats, such as immunoglobulin and T-cell subtype CD4+, in serum are normalized.	(125)
	Polysaccharides	*In vivo* immunity	Broiler (14 days age)	Enhancing lymphocyte multiplication, boosting interferon-γ and interleukin-2, and increasing the antibody titer in serum	(126)
**Aqueous garlic-lemon** **extract**	Allicin	*In vivo* anticancer	Female albino mice	Releasing liver injury through glutathione and total protein levels, as well as the elevation of AST, alkaline phosphatase, and alanine aminotransferase levels	(127)
	DADS	*In vivo* anti-cancer	FVB/N mice	Preventing the development of colorectal tumors produced by azoxymethane and dextran sulfate	(128)
	SAMC	*In vivo* anticancer	Hepatoma xenograft model in mice Huh-7 nuclear	Reducing cell viability through cell membrane interaction with the Wnt-pathway co-receptor LRP6	(104)
**Garlic-clove extract**	Group of active compounds	*In vivo* hepatoprotective	Male rabbits	Maintaining the liver cells against CCl_4_ injury	(129)
**LABFGE**	DADS	*In vivo* hepatoprotective	Wister rats	Reducing fatty liver through a long-term high-fat diet by boosting antioxidants by reducing cytochrome P450 2E1 expression	(130)
	Garlic oil	*In vivo* hepatoprotective	1,3-Dichloro-2-propanol-challenged rats	Increasing antioxidant enzyme activity in the liver, inhibiting the metabolism of 1, 3-dichloro-2-propanol, Reducting liver apoptosis	(131)
**Aqueous garlic extract**	Group of active compounds	*In vivo* renal protection	Diabetic rats	Mitigating oxidative stress in the kidney	(132)
	DATS	*In vivo* renal protection	Albino rats	Enhancing the biochemical components in renal plasma mediated by alloxan	(133)
	Group of active compounds	*In vivo* renal protection	Wister rats	Mitigating oxidative stress in the kidney induced by As	(109)

LABFGE, lactic acid bacteria fermented garlic extract; DAS, diallyl sulfide; DATS, diallyl trisulfide; DADS, diallyl disulfide; SAMC, sallylmercaptocysteine; SAC, S-allyl cysteine; and SPRC, S-propargyl-L-cysteine.

### 6.1 Antimicrobial activity of garlic

The minimum inhibitory concentration (MIC), minimum sensitivity, and minimum bactericidal concentration (MBC) assays are frequently employed to quantify and communicate the antimicrobial properties of garlic and allicin (134). The antibacterial attributes of allicin found in garlic have been acknowledged for a considerable duration. Extensive research was conducted between 1944 and 1970 on bioeffects of garlic. These studies revealed that garlic extract exhibited exceptional antibacterial properties against Gram-positive and Gram-negative bacteria and specific medically significant fungi (135, 136). A comprehensive understanding of allicin’s antibacterial properties is still elusive; nevertheless, it has been established that it influences the free amino acid L-cysteine by generating allyl-disulfide species (136). Using the microdilution method, an *in vitro* study examined the antibacterial properties of synthetic allicin against 38 isolates of methicillin-resistant and methicillin-sensitive staphylococci (137).

Perez-Giraldo et al. (137) also assessed the effect of allicin on biofilm formation. The outcomes were indistinguishable between methicillin-sensitive and methicillin-resistant bacteria, and it was determined that all cultures examined demonstrated considerable sensitivity to allicin, as indicated by MIC values ranging from 0.25 to 16 mg/L. In an *in vitro* study using five strains of *Staphylococcus epidermidis*, it was reported that adding half the MIC of allicin substantially affected strain proliferation and biofilm formation (137). According to these findings, allicin might play a beneficial function in preventing the attachment of these microorganisms to medical equipment (137). The administration of allicin (128 µg/mL) substantially impacts the synthesis of extracellular polysaccharide components and their ability to adhere to surfaces, compared to a saline control group (138).

Allicin is also notable for its ability to prevent the growth of *Pseudomonas aeruginosa* biofilms and the quorum-sensing-regulated generation of virulence components by reducing the expression of specific enzymes and toxins (elastase and exotoxin A) (139). Thus, allicin may be a viable option for inhibiting the formation and development of *P. aeruginosa* biofilms (139). Research conducted by Shuford et al. (140) evaluated the efficacy of garlic extract as an anti-biofilm agent against both the sessile and planktonic strains of *Candida albicans*. They reported that adding fresh aqueous garlic extract may inhibit *C. albicans* biofilm development during the planktonic, adhesive, and sessile phases. Zhai et al. (141) analyzed the application of allicin with anti-biofilm properties. A scanning electron microscopy investigation into the impact of allicin and vancomycin on the growth of *S. epidermidis* biofilms was identified as the cause of aggregation and biofilm formation (141). In contrast to the individual effects of allicin and vancomycin, the results demonstrated that the combined effect of the two agents inhibits *S. epidermidis* growth, attachment, and biofilm formation on the outermost layer of a prosthesis (141).

The potential of garlic and its sulfur constituents as antiviral agents has been demonstrated *in vivo*. Globally speaking, the 2019 coronavirus disease epidemic (COVID-19) is the most critical public health crisis. As a result of the social distancing measures imposed by COVID-19, psychological, financial, and social welfare have undergone substantial transformations in recent years. Lung tissue inflammatory responses and pro-inflammatory pathways (such as IL-6 and IL-1) are primarily activated by the severe acute respiratory syndrome coronavirus 2 (SARS-CoV-2)-caused COVID-19 pandemic (142). Viral expression of the angiotensin-converting enzyme 2 (ACE2) receptor can potentially induce harm to critical organs and tissues.

Furthermore, the dysregulation of the ACE2/angiotensin-(1–7)/mas axis and the renin-angiotensin system after SARS-CoV-2 infection worsens comorbidities and multi-organ lesions (143). A cure for the fundamental ailment that has initiated this epidemic remains unknown. Drugs such as hydroxychloroquine, remdesivir, umifenovir, favipiravir, ribavirin, lopinavir/ritonavir, and hydroxychloroquine, among others, may be administered as therapeutic interventions unless explicitly prohibited. In addition to dietary therapy and herbal medication, supportive treatments may effectively prevent the spread of COVID-19 (142, 143).

Certain foods and herbs contain bioactive substances with antioxidant, antimicrobial, and immunomodulatory properties that may enhance the function and quantity of natural killer cells, macrophages, lymphocytes, and cytokine suppressors (144). As a result, these substances may aid in pre- and post-exposure prophylaxis. As a result, herbal remedies alleviate the adverse effects of antivirals by reducing the necessary dosage and synergistically improving treatment and outcomes by reducing respiratory symptoms and inflammatory reactions (144). The numerous health benefits of garlic can be attributed to its sulfur-containing phytochemicals. These include preventing cardiac disease, regulating the immune system, lowering blood sugar levels, combating cancer, and reducing inflammation. The most prevalent sulfur compounds in garlic account for 82% of the total. These include allicin, diallyl (di and tri-) sulfide, ajoenes (E- and Z-ajoene), S-allyl cysteine sulfoxide, and vinyldithiins (2-vinyl-(4H)-1,3-dithiin, 3-vinyl-(4H)-1,2-dithiin). Garlic, apart from alliin, is known to comprise SAC, N-acetylcysteine, and S-allyl-mercapto cysteine, among other organosulfur compounds (145). Garlic has been found to possess antiviral properties against a range of viruses, including influenza B, HIV (type 1), herpes simplex virus (types 1 and 2), vesicular stomatitis virus, coxsackievirus, and gammaretrovirus (146).

Recent research has identified the positions of specific amino acids (Thr26, Asn119, Thr24) in the enzyme’s active site of the serine-like protease Mpro [chymotrypsin-like protease (3CLpro)] of SARS-primary CoV-2 (e.g., 2GTB and 6 LU7) (147). A significant degree of structural homology (96.0%) was observed between Mpro from SARS-CoV types 1 and 2. By impeding viral polyprotein cleavage, SARS-CoV-2 infection rates can be significantly diminished because viral polyprotein cleavage is essential for effective protein synthesis and viral replication (147).

In an *in silico* analysis of the inhibitory activity of garlic, seven onion-specific compounds (OSCs) were identified as resistant to SARS-CoV-2 (148). These compounds included S-allymercapto-cysteine, S-propyl-L-cysteine, S-(allyl/methyl/ethyl/propyl)-cysteine, and alliin. A molecular docking investigation demonstrated that, among the various cysteine sulphoxides, garlic alliin exhibited the most potent antiviral activity against COVID-19. SARS-CoV-2 could be eradicated by utilizing this bioactive component in isolation or combined with the widely recognized primary treatment medication (148).

The phytochemicals of black pepper, black cumin, and ginger exhibited a comparable inhibitory effect upon isolation (148). Garlic clove extract exhibited a significant inhibitory effect on the growth of SARS-CoV-1 *in vivo* at a concentration of 0.1 mL. This effect was likely mediated by the extract’s ability to impede the synthesis of genetic material and structural proteins (149). By inhibiting the protease found in SARS-CoV-1, quercetin might also impede the viral attachment phase of replication (150). Flavonoids (e.g., quercetin) and organosulfur compounds (e.g., allicin) present in aqueous extracts and essential oils of garlic may account for the decreased viral infection rate induced by SARS-CoV-2 through their interaction with the protease Mpro. Encapsulation is a technique to safeguard bioactive compounds’ functionality and oxidative stability (149, 150). Simultaneously, it permits the controlled release and distribution of these compounds to their designated sites of action within the body in the form of medication particles measuring microns or nanometers in size. Finally, to mitigate the transmission of COVID-19 among specific populations, it may be necessary to restrict their intake of foods containing free or encapsulated garlic bioactive ingredients to a moderate degree (150).

### 6.2 The potential anti-inflammatory effects of garlic

Garlic and its derived beneficial components have been found to induce suppression in numerous physiological disorders, such as cardiovascular complications, oxidative stress, cancer proliferation, and immunological dysfunction. NO_2_ and prostaglandin E-2 production were suppressed in lipopolysaccharide-stimulated RAW 264.7 macrophages by garlic ethyl linoleate (83). This was achieved through the downregulation of inducible NO synthase expression and the upregulation of heme oxygenase-1 expression. Garlic’s potential utility lies in its ability to augment natural killer cells, stimulate interferon-gamma synthesis, tumor necrosis factor-alpha, and interleukin-2, and counteract the immune response suppression associated with an elevated cancer risk (151). Furthermore, allicin can potentially mitigate the inflammatory response initiated by schistosome infection in BALB/c rodents, thus establishing itself as a feasible adjuvant therapy (84).

**Figure 3:**
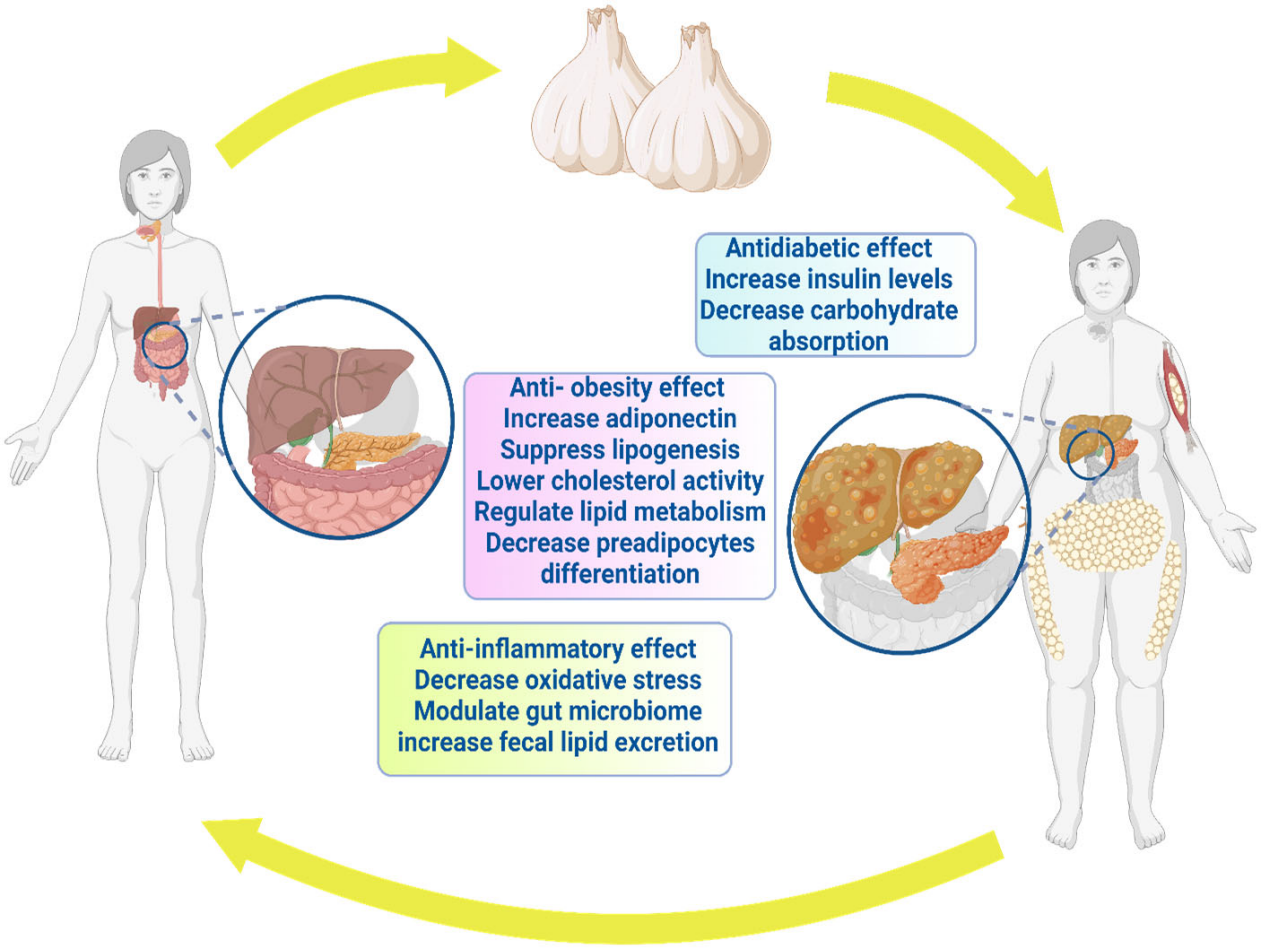
Antiobesity, antidiabetic and anti-inflammatory effects of garlic.

Furthermore, several research studies have indicated that individuals who are overweight or obese and suffer from osteoarthritis may benefit from garlic supplements as they reduce their resistance (85). Therefore, garlic effectively inhibited inflammatory pathways in both *in vitro* and *in vivo* investigations through the reduction of inflammatory mediators (including interleukin (IL)-1, tumor necrosis factor-alpha (TNF-α), and NO). Garlic exhibits potential as a therapeutic intervention for inflammatory conditions, including arthritis in humans, since its toxicity is minimal or non-existent (151). **Figure 3** depicts the anti-inflammatory effects of garlic.

### 6.3 Garlic potential as an antioxidant and anticancer agents

According to a growing body of research, garlic exerts potent antioxidant effects. Aged garlic extract possesses greater antioxidant properties than fresh garlic and other available garlic supplements (28). The antioxidant activity of water-soluble organosulfur compounds such as SAC and SAMC is high. SAC and SAMC are the principal organosulphur components of aged garlic extract (152). DADS, DAS, and diallyl trisulfide are fat-soluble allyl sulfides with antioxidant potential (153). Raw garlic showed greater antioxidant activity than cooked garlic, according to a study that examined the antioxidant capabilities of raw and cooked garlic (154). The antioxidant capacity of stir-fried garlic was also greater (carotene bleaching), indicating that processing may affect the garlic’s antioxidant properties (154).

The ethanolic extract possesses more robust antioxidant activity than raw garlic, 2,2-diphenyl-1-picrylhydrazyl (DPPH), and oxygen radical absorption capacity (ORAC) confirmed these results (155). Additionally, DPPH, 2,2'-azino-bis-3-ethylbenzothiazoline-6-sulfonic acid (ABTS), ferric reducing antioxidant power, H_2_O_2_ scavenging, and Fe^2+^ chelating assays revealed that aged garlic extract possessed greater antioxidant properties than fresh garlic (156). Interestingly, single-clove garlic extract contained a higher concentration of phenolic compounds and exhibited more antioxidant activity than multi-clove garlic extract (129). In addition, heat treatment improved the antioxidant activity of black garlic, with the peak antioxidant capacity occurring on the 21^st^ day of processing (157, 158). Moreover, the increased pressure enhanced the garlic paste’s antioxidant qualities (159). During fermentation, however, “Laba” garlic, a traditional Chinese garlic product, loses its antioxidant properties (160). Garlic saponins have been found to prevent H_2_O_2_-induced growth inhibition and DNA damage in C_2_C_12_ myoblasts generated from mice (78).

Garlic and its active constituents (phenols and saponins) are antioxidants (161). Various processing procedures also modulated the antioxidant activity of garlic (45). The antioxidant activity of raw garlic was greater than that of cooked garlic, and the antioxidant activity of raw garlic was more than that of cooked garlic (154). The potency of fermented garlic, such as black garlic, is greater than that of raw garlic (162). In addition, the cellular experiment demonstrated that the mechanism of garlic’s antioxidative effect might include the modulation of the Nrf-2-ARE pathway and the augmentation of antioxidant enzyme activity (78).

Even though cancer is a significant contributor to mortality worldwide, several spices and foods including ginger, berries, tomatoes, and cruciferous vegetables have been shown to possess potent anticancer properties (163). Recent research has demonstrated that garlic and its bioactive constituents inhibit the development of numerous types of cancer, including those of the gastrointestinal tract, colon, lungs, and urinary tract (102). Daily, individuals frequently encounter numerous carcinogenic substances (164). According to an *in vitro* study, garlic’s sulfur-containing compounds inhibited the activation of cancer-causing substances (165). Furthermore, the production of nitrosamines, typical carcinogens generated during heating and storage, could be inhibited by garlic and the organic allyl sulfides it produces (166).

Furthermore, the allyl sulfides in garlic inhibit DNA alkylation, an early step in developing nitrosamine carcinogenesis (167). Cancer cells are primarily characterized by their uncontrolled proliferation, a distinctive feature of the ailment. Previous studies (168) have documented the high sensitivity of various human cancer cell lines to the antiproliferative effects of unrefined garlic extract, including colon (Caco-2), prostate (PC-3), breast (MCF-7), and liver (HepG2) cancer cells (168). By inhibiting the phosphorylation of Cdc25C and Cdc2, they are increasing the expression of p21WAF1 and decreasing the phosphorylation of Cyclin B1 (168, 169). Garlic extract (107, 170) improved checkpoint kinase two and G2/M-phase cell modification. Research has demonstrated that whether in its unprocessed or ground state, garlic can upregulate the activity of apoptosis-related genes, including hypoxia-inducible factor 1, proto-oncogene c-jun, and aryl hydrocarbon receptor (118, 171).

Additionally, the aged garlic extract inhibited the motility, development, and tube formation of ECV304 endothelial cells, altering the cellular composition of rat lungs (79). Therefore, garlic and its bioactive constituents have the potential to serve as a beneficial strategy in the prevention and management of specific types of cancer (172, 173). Primary anticancer strategies include initiating apoptosis, inhibiting the growth and division of cancer cells, regulating the metabolism of carcinogenic agents, limiting the formation of new blood vessels, and minimizing invasion and metastasis (174, 175).

**Figure 4:**
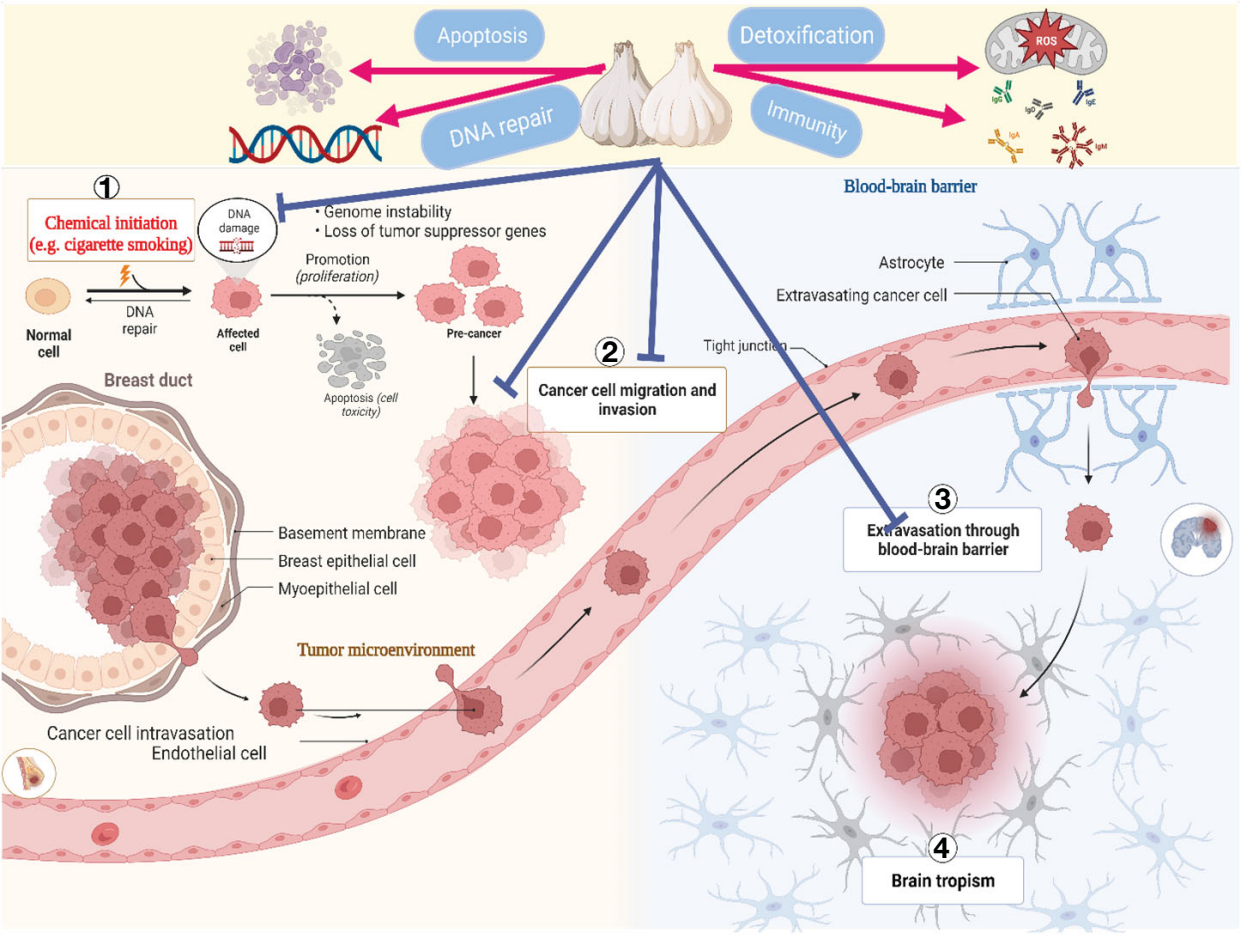
The anticancer mechanisms of garlic-derived phytochemicals.

**Figure 4** illustrates the process by which garlic’s bioactive components inhibit the growth of cancer cells.

### 6.4 The potential immunomodulatory effects of garlic

Garlic and its derived bioactive constituents, including organosulfur compounds, may enhance immune system performance via pathways such as oxidative stress, interleukins, immunological and inflammatory response, and others, according to numerous *in vitro* and *in vivo* experimental studies (176). Upon exposure to polysaccharides derived from garlic, RAW 264.7 macrophages exhibit distinct variations in tumor necrosis factor-α, IL-10, IL-6, and interferon-γ expression. These polysaccharides possess notable immunomodulatory properties (177).

Fresh garlic polysaccharides exhibit a more pronounced immunomodulatory impact than black garlic polysaccharides (93). This effect results from fructan degradation associated with the process (177). In an *in vivo* study, Li et al. (177) showed that by administering garlic oil to Wistar rats 30 min before diazinon, a range of immunological parameters were restored to normalcy. These parameters included the serum total immunoglobulin level and T-cell subtype CD4+. Additionally, research has demonstrated that administering aged garlic extract to individuals may reduce the severity of specific viral infections, such as the common cold, and strengthen the immune system (93). This indicates that garlic’s primary immune-modulating components are polysaccharides (93).

## 7 Therapeutical application of bioactive compounds in garlic

### 7.1 Anti-hypertensive function

Garlic may decrease oxidative stress, inhibit the angiotensin-converting enzyme, and increase the production of NO and H_2_S, all of which contribute to blood pressure reduction (178, 179). By increasing NO production, aged garlic extract induced endothelial-dependent vasodilation in individual rat aortic rings, according to one study. Moreover, L-arginine was crucial for NOS-mediated NO production in aged garlic extract (178). Additionally, it was demonstrated that S-1-propylene cysteine was the principal antihypertensive component in the aged garlic extract. It was demonstrated that administration of S-1-propylenecysteine decreased the systolic blood pressure of rats with spontaneous hypertension but had no effect on control rats (180). In an additional investigation, nitrites present in fermented garlic extract (FGE) were metabolized to NO through the action of *Bacillus subtilis* (180).

Furthermore, NO decreased systolic blood pressure in rodents with spontaneous hypertension via the protein kinase G (PKG)-soluble guanylyl cyclase (sGC)-cyclic guanosine monophosphate (cGMP) signaling pathway (181). By downregulating vascular endothelial cell adhesion molecule-1 and matrix metalloproteinase-9 (MMP-9) and upregulating PKG and endothelial nitric oxide synthase (eNOS), FGE decreased blood pressure in rats with pulmonary hypertension induced by monocrotaline (181). Furthermore, the efficacy of captopril in reducing hypertension and angiotensin-converting enzyme (ACE) in rodents was improved through the combination of captopril with garlic and its bioactive component, alliin (182). Systolic and diastolic blood pressures of 44 hypertensive participants registered for a double-blind, placebo-controlled study experienced a substantial reduction when garlic was prepared with enzymatic browning (183).

### 7.2 The impact of garlic on kidney and liver functions

Recent *in vivo* studies have demonstrated garlic’s effective reduction of nephrotoxicity (184). After being exposed to garlic’s aqueous component, the kidneys of diabetic rats showed a reduction in oxidative stress (132). In addition, the water-based element of garlic improved the levels of renal plasma biochemical markers in Wistar rat models that were affected by alloxan (109). In addition, studies have shown that diallyl trisulfide can activate the Nrf2-ARE pathway in rats, which helps protect kidney tissue from oxidative stress caused by arsenic (174).

Numerous studies have indicated that various natural compounds, such as garlic, can positively impact liver function (185). In an *in vivo* study, researchers discovered that a natural extract derived from black garlic significantly reduced the harmful effects of tert-butyl hydroperoxide on rat clone-9 hepatocytes (112). This extract effectively countered oxidative stress, lipid peroxidation, inflammation, and apoptosis, showcasing its potential as a valuable therapeutic agent. Ascorbic acid and garlic effectively prevented liver damage caused by Cd in an albino mouse model (112). An *in vivo* study using a male rabbit model found that a single clove of garlic provided more excellent protection against acute liver damage induced by CCl_4_ compared to multiple cloves of garlic (129).

Furthermore, it has been proposed that the active compounds found in garlic, specifically S-methyl-l-cysteine, DADS, and DAS may have potential applications in treating and preventing liver damage caused by ethanol. A study found that DADS in garlic essential oil reduced the effects of nonalcoholic fatty liver disease caused by a high-fat diet in rats (186). Through its effects on cytochrome P450 2E1, DADS enhanced antioxidant capacity and decreased pro-inflammatory cytokine production in liver tissue (186). Lai et al. (130) reported that fermented garlic extract, shows potential as a treatment for chronic hepatic diseases (130).

In a study by Kim et al. (187), a randomized, double-blind, placebo-controlled trial was conducted to examine the impact of fermented garlic extract on hepatic activity in 36 individuals with moderately elevated blood gamma-glutamyl transpeptidase concentrations. Based on the results, no unexpected outcomes were linked to the increased alanine aminotransferase levels and gamma-glutamyl transpeptidase. Therefore, garlic has the potential to treat either condition effectively.

### 7.3 The impact of garlic on the digestive system

Garlic has been extensively studied for its positive impact on digestive processes. In a study conducted in a laboratory setting, it was found that black garlic extract has the potential to enhance bowel movements. This is achieved by promoting bowel emptying and stimulating peristaltic action. An *in vitro* study found that the aquatic fraction of black garlic had a more significant impact on improving gastrointestinal function in the small intestine than the n-butanol and ethyl acetate mixture (116). Additionally, therapy involving cabbage and garlic extract showed a decrease in the severity of gastric ulcers and a reduction in gastric juice volume, stomach acid levels, microbial count, and histological alterations. This type of treatment improved stomach acidity in rat models (188).

*In vivo* rat models, aged garlic extract effectively reduced the development of ulcers caused by indomethacin. This was achieved by increasing the levels of prostaglandin E-2 and glutathione while also reducing oxidative stress in the stomach tissue of the rats (90). Like a pharmacologist, DADS was found to suppress the expression of signal transducer and transcription 1 (STAT-1) activator in interferon-induced intestinal cells (117).

Additionally, DAS was observed to suppress the expression of interferon-inducible protein-10, interleukin-6, and nitric oxide. In a mouse model, the colitis caused by dinitrobenzene sulfonic acid was made worse by including DAS and DADS (117). Raw garlic has been shown to positively impact microbial urease function and *Helicobacter pylori* levels in the stomachs of patients (121).

### 7.4 The impact of garlic on cardiovascular functioning

In recent years, there has been a significant increase in the death rate from cardiovascular illnesses (189). There has been a recent surge in interest in utilizing natural crops to support optimal heart health, and garlic stands out as one of the most promising choices (190). Eating garlic powder has been shown to reduce several risk factors for cardiovascular disease, including total cholesterol, blood pressure, and low-density lipoprotein cholesterol (191).

Garlic has the potential to offer various health benefits in this field. These include an increase in the production of NO and H_2_S, a reduction in oxidative stress, and the ability to inhibit the angiotensin-converting enzyme, which can help lower blood pressure (117). Research conducted by Takashima et al. (178) suggests that aged garlic extract may have the potential to stimulate NO synthesis and promote endothelial-dependent vasodilation in the aortic rings of rats. It was found that the L-arginine in aged garlic extract plays a crucial role in NOS-mediated NO production (117).

Garlic has the potential to support the maintenance of the heart. Garlic when combined with allicin, the rat model showed enhanced antihypertensive and ACE-lowering effects of captopril (182). In an animal model of insulin resistance and obesity, garlic extract effectively reduced cardiac and mitochondrial dysfunction (192). When allicin comes into contact with thiols, it transforms organic diallyl polysulfide. This compound plays a crucial role in supplying H_2_S to support the proper functioning of the heart (193). Given these discoveries, garlic has been the focus of numerous clinical trials exploring its potential in treating hyperlipidemia, hypertension, and cardiovascular disease. Garlic may exert its effects through various mechanisms, including modulation of NO and H_2_S production, reduction of oxidative stress, and inhibition of angiotensin-converting enzyme activity (193).

### 7.5 Anti-diabetic activity

Regarding diabetes, garlic prevented pancreatic cell damage and associated pathological alterations (194). Moreover, diabetes-linked retinopathy risk was significantly reduced through garlic (195). Al-Brakati (119) addressed significant improvement in retinal tissue after seven weeks of oral administration of raw garlic. Moreover, Thomson et al. (196) noticed the favorable modulatory effect of garlic on body weight and blood glucose level. Aged garlic extract pointed out that antidiabetic behavior correlated with dosage (196). Garlic supplements showed reduced fructosamine and glycosylated hemoglobin in type 2 diabetes patients (174). Consistently, garlic has shown potential in treating type 2 diabetes (197). In conclusion, garlic can be a candidate for protecting and treating diabetes and diabetic disorders (198). **Figure 3** depicts the antidiabetic benefits of garlic.

### 7.6 Anti-obesity activity

Garlic and sulfur-containing compounds can help lose weight by inhibiting 3T3-L1 adipocytes and acetyl-CoA carboxylase (ACC-1) (120, 199). Lee et al. (200) addressed an activation in adenosine monophosphate-activated protein kinase (AMPK)I in addition to carnitine palmitoyl transferase (CPT-1). Consistently (201), found an increase in UCP-1 through Kruppel-like factor 15 (KLF15), linked to brown adipocytes that represent a crucial phenotype for protection against obesity. Furthermore, in the diet-induced obesity model, fermented garlic (250 and 500 mg/kg) revealed lowered body weight with underlying decreased cholesterol levels. Garlic oil reduced body weight and white adipose tissue quantity in the high-fat diet model (201). El-Demerdash et al. (202) observed an improved O_2_ consumption during the night with interesting increased energy expenditure during the daytime. Similarly, garlic extract in water was linked to hypoglycemic impact (202).

Sanie-Jahromi et al. (203) noted significant hypoglycemic impact among garlic compounds, alliin, allyl sulfide, and glibenclamide (203). Garlic oil (10 mg/kg i.p.) and diallyl trisulfides promoted both sensitivity and secretion of insulin in diabetic rat models (28, 202, 204). In high-fat diet mice, lactic acid bacteria fermented garlic extract (LABFGE) showed an anti-obesity property with significantly decreased body weight and adipose tissue (113). LABFGE's ability to reduce lipogenesis is attributed to a decrease in the expression of lipogenic proteins (113). Methanolic extract of black garlic (MEBG) reversed the weight gain of a high-fat diet (205). MEBG modulated the network of adipogenesis, adipokine biosynthesis, and lipolysis, such as upregulation of AMPK, FOXO1, and adiponectin, whereas MEBG downregulated CD36 and TNF-α in HFD rats (115). Therefore, we could address that garlic and garlic-derived compounds have a potential role as anti-obesity and can reduce lipogenesis. The anti-obesity properties of garlic are illustrated in **Figure 3**.

### 7.7 Cardiovascular and neuroprotection

In recent years, cardiovascular disease has been the leading cause of fatality (190, 191). Recently, interest in using natural crops to support cardiac health has increased, and garlic is among the most promising candidates (190). Several cardiovascular disease risk factors, including low-density lipoprotein cholesterol, total cholesterol, and blood pressure, are reduced by consuming garlic powder (194). Potential prospective health benefits of garlic in this domain include the following: (1) increased production of NO and H_2_S; (2) reduced oxidative stress; and (3) inhibition of the angiotensin-converting enzyme, which ultimately results in reduced blood pressure (117).

Takashima et al. (178) conducted research suggesting that administering matured garlic extract to rodents could induce endothelial-dependent vasodilation in the aortic rings by stimulating NO synthesis. In addition, the L-arginine in aged garlic extract was found to be crucial for NOS-mediated NO production (178). Furthermore, adding garlic and allicin enhanced captopril’s antihypertensive and ACE-lowering effects in the rodent model (182). An additional organ that garlic may aid in maintaining is the heart. Cardiovascular and mitochondrial dysfunction were mitigated by garlic extract in an animal model of obesity and insulin resistance (192). Allicin was readily converted to organic diallyl polysulfide upon exposure to thiols; this compound supplied H_2_S to sustain the heart’s activity (203). In light of these findings, garlic has been the subject of numerous clinical trials regarding its potential to treat hyperlipidemia, hypertension, and cardiovascular diseases (203). Potential principal mechanisms of action for garlic include alterations in the production of NO and H_2_S, a reduction in oxidative stress, and a decrease in the activity of angiotensin-converting enzyme (203).

It was reported that an aged garlic extract-carbohydrate derivative, N-α-(1-deoxy-D-fructos-1-yl)-L-arginine, could reduce neurological inflammation in a mouse model of BV-2 microglial cells activated lipopolysaccharide by regulating the expression of numerous oxidative stress-related protein targets and inhibiting NO production (192, 193). Ho and Su (206) suggested that the anti-neuritis effect of garlic against lipopolysaccharide-activated BV-2 microglial cells (206) was due to its organosulfur components. Supplementation with garlic in pregnant and lactating rats reduced blood and brain Pb concentrations and prevented Pb-induced neuronal loss during hippocampal development (207).

Utilizing the Basso, Beattie, and Bresnahan (BBB) scoring system, the neurological impact of aging on a spinal cord I/R model of rats was evaluated (208). Significantly higher BBB scores were observed in the aged garlic extract group compared to the I/R group, suggesting that aged garlic extract exerted a substantial neuroprotective effect. Aged garlic extract also increased levels of glutamate decarboxylase and vesicular glutamate transporter 1 in the hippocampus, which enhanced working memory in rodents, additionally, it reduced the loss of cholinergic neurons (208).

**Figure 5:**
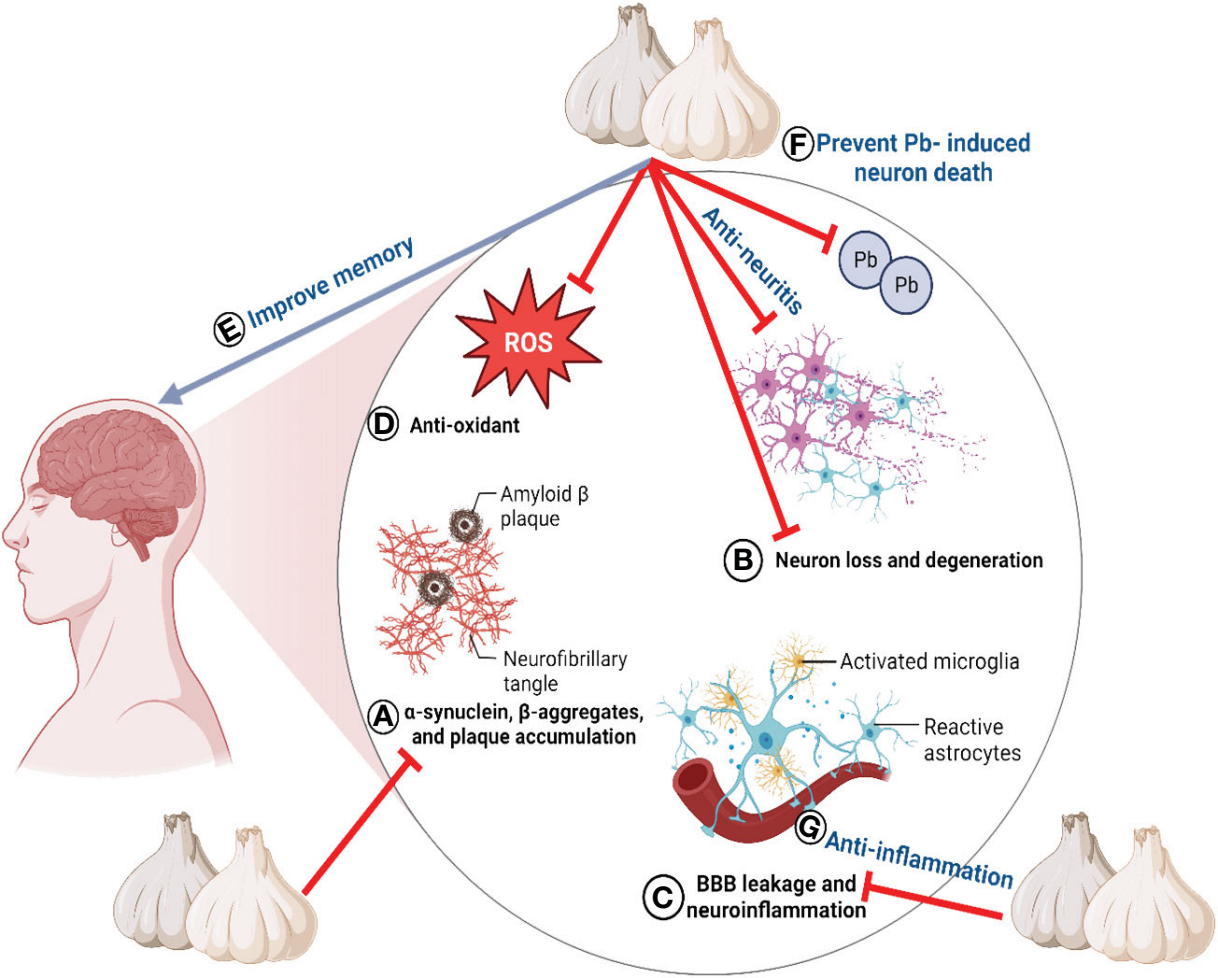
The neuroprotective properties of garlic. All lables **(A–G)** are the sequence of events/properties of garlic toward neuroprotection.

Furthermore, research has demonstrated that a garlic ethanol extract can enhance memory (88). Garlic treatment increased the concentrations of Na^+^/K^+^ ATPase, Ca^2+^ ATPase, and glutamine synthetase in the brains of diabetic Wistar rats (209). In addition, the detrimental effects of monosodium glutamate on working memory were effectively counterbalanced by the fermented garlic ethanol extract (210). Z-ajoene has demonstrated advantageous effects in the CA1 region of the hippocampus by decreasing lipid peroxidation and mitigating delayed neuronal mortality and gliosis induced by I/R stimulation (111). SAC ameliorated rat cognitive impairment due to reduced acetylcholinesterase activity, neuroinflammation, astrogliosis, and oxidative stress (110). The neuroprotective effects of garlic primarily target the hippocampus, as demonstrated by both *in vivo* and *in vitro* studies. Substantial evidence indicates that organic sulfur compounds play a vital function in protecting neurons (110). The neuroprotective properties of garlic are illustrated in **Figure 5**.

## 8 Safety of garlic bioactive compounds and their derivatives

### 8.1 Suggested dosage

The elderly are advised to consume four grams of fresh garlic, seven and a half grams of matured garlic extract, or one dried garlic powder tablet three or four times daily (211). In their study, Rana et al. (212) demonstrated that administering garlic orally or intraperitoneally to rats at a 50 mg/kg dose had no discernible impact on liver and lung tissue. However, rats consuming garlic daily at doses of 250, 500, and 1000 mg/kg developed acute alterations in liver and lung tissue, indicating the presence of dose-dependent toxicity (212). Upon light microscopy and ultrastructural analysis, morphological abnormalities in the liver were observed at a daily dosage of 1000 mg/kg garlic. In contrast, auto-antioxidant levels were significantly reduced at 500 and 1000 mg/kg garlic concentrations without affecting lipid peroxidation levels. Histological analysis further demonstrated the presence of nonspecific focal injury on hepatocytes (212).

A study by Mikaili et al. (213) demonstrated that biological and histological markers in male and female rodents were altered, and development was halted at 300 and 600 mg of garlic bulb extract. Asdaq and Inamdar (214) showed that the combination of 250 mg/kg of garlic and hydrochlorothiazide offers cardioprotective and synergistic effects against fructose and isoproterenol toxicity. Antioxidant enzyme activity was substantially increased in the presence of propranolol and 250 mg/kg garlic during ischemia-induced injury (214).

### 8.2 Hazards and adverse reactions

The US Food and Drug Administration (FDA) has determined that garlic is safe for human consumption; however, sensitive stomachs may experience distress when consuming large quantities of garlic. Randomized controlled trials examining the safety of garlic documented the following adverse effects: insomnia, dizziness, emesis, tachycardia, headache, flushing, mild orthostatic hypotension, sweat, discomfort, foul body odor, and defecation (212). When consumed in excess on an empty stomach, fresh garlic can cause intestinal flora to shift, flatulence, and bloating. The topical application of fresh garlic also led to the development of excruciating lesions, rashes, and burns (215).

In contrast to the prevailing notion that garlic does not affect medication metabolism, recent studies involving healthy volunteers indicated that its antithrombotic properties might result in an inconsistent effect of garlic on the pharmacokinetics of anticoagulants and protease inhibitors (216). As it has been shown to prolong bleeding in one individual with an epidural spontaneous hematoma, many surgeons advise against consuming large quantities of garlic 7–10 days before surgery (216).

Long-term, high-dose exposure to raw garlic in animals results in anemia and weight loss due to the lysis of red blood cells (RBCs), whereas 5 mL/kg of raw garlic liquid injected into animals induces gastrointestinal damage and mortality. Spermatogenesis is inhibited in rodents when 50 milligrams of garlic powder are administered daily for an extended period (212). This results in decreased sialic acid levels in the testes, the bladder, and seminal vesicles and decreased Leydig cell activity (212). As evidenced by the formation of Heinz bodies in RBCs and the development of methemoglobinemia, oxidative hemolysis is the principal toxicological mechanism by which sulfur compounds derived from *Allium* (217). Initial clinical manifestations included depression, appetite loss, vomiting, abdominal discomfort, anemia manifested by pallid mucous membranes, diarrhea, jaundice, lethargy, accelerated heart and respiratory rates, and hemoglobinuria (218). The onset of symptoms associated with garlic poisoning can vary significantly, spanning from one day to several weeks following ingestion (218).

Anticoagulant and fibrinolytic properties, in addition to a significant decrease in platelet production and conflicting effects on fibrinolytic effectiveness, have been documented in garlic, as reported in prior studies (219). Upon oral ingestion, dehydrated raw garlic powder damages the stomach mucosa promptly, as demonstrated by Chen et al. (220). However, experimental mice’s intestinal mucosa is protected by the sulfur-free compound aged garlic extract, as reported by Yüncü et al. (221). Research studies have established that while garlic is deemed safe when consumed in moderation amounts, therapeutic doses may induce gastrointestinal distress, and excessive quantities have been linked to liver harm (188). Allicin’s potential toxicity could be attributed to its capacity to traverse cellular membranes and interact with cellular thiols, such as glutathione or cysteine residues in proteins (132), as well as enzymes incorporating reactive cysteine (222). Rana et al. (212) discovered that allicin or garlic powder at a concentration of 200 mg/mL could potentially cause severe cell injury in the livers of isolated rats (212).

## 9 Food application of garlic bioactive compounds and their derivatives

Food illness and decay may result from pathogenic bacteria or other microorganisms. Antibiotics are frequently employed in the fight against these microorganisms. Antioxidants are gaining popularity as a method of preventing nutrient losses and extending the storage life of foods by preventing oxidation. However, excessive antibiotic use may lead to the emergence of antibiotic resistance (142). The majority of artificial antimicrobial agents authorized for use as food preservatives by regulatory bodies, based on studies conducted by Gutiérrez-del-Ro et al. (223), pose a risk to consumer health.

Sulfites, a class of thiol compounds used as commercial food preservatives, have been associated with numerous adverse nutritional effects, including ingesting thiamine, commonly referred to as vitamin B1. Therefore, preservatives derived from naturally generated antimicrobial compounds discovered in microorganisms, plants, and animals are widely employed. Antimicrobials prevent the spoilage of food and impede the transmission of disease (224).

Many individuals use natural and safer preservatives to impart an “organic” or “green” appearance to their food. Investigations have been directed toward the potential use of essential oils derived from botanicals as food preservatives (225). Garlic in food storage extends the expiration life by six days when refrigerated and slows the rate of food spoilage caused by microorganisms, according to recent studies (226). The utilization of garlic paste extended the freshness of Nile tilapia by over three days, according to research conducted by Kombat et al. (227).

Research on extending the shelf life of ready-to-eat (RTE) products has primarily focused on utilizing natural organic essential oils (EOs) and coatings. The extraction of plants EOs results in low harvests and high costs (228, 229). Therefore, in culinary applications, plant aqueous extracts may function as acceptable alternatives to natural essential oils (230). Diao et al. (231) investigated the preservation effect of carboxymethyl chitosan (CMCS) ultrasonicated coating modified with garlic aqueous extracts on RTE fragrant poultry. By employing ultrasonic processing, CMCS, and garlic aqueous extracts were transformed into a rudimentary nano-coating solution that satisfies the safety requirements for human consumption. Utilizing the ultrasonicated coating method devised with garlic aqueous extracts-CMCS may be advantageous for RTE poultry meat (231).

Garlic essential oil derives its antibacterial properties from its substantial concentration of organosulfur compounds. Primarily present in garlic essential oil, allicin is susceptible to degradation when exposed to acidity and temperature shocks. Encapsulation may enhance the stability and solubility of the substance in water-based foods. Additionally, encapsulation obscures the foul, pungent odor of pure garlic EOs (232). A bioactive component in liquid, solid, or gaseous state is encapsulated within a matrix of inert material via nanoencapsulation to maintain coated items. Nanoencapsulation can potentially enhance the sensory attributes of products through the augmentation of bioactive chemical stability and the obfuscation of offensive flavors and aromas (233).

Phospholipids and naturally active phytochemicals that are structurally bound are produced when phosphatidylcholine (or another hydrophilic polar head group) reacts with plant extracts in an aprotic solvent to form phytosomes. The findings of Nazari et al. (58) indicated that garlic essential oil nanophytosomes have the potential to exhibit desirable physicochemical characteristics, particularly when incorporated into acidic food products (58). Youssef et al. (234) also created bio-nano composites from Arabic gum (AG), carboxymethyl cellulose (CMC), titanium dioxide (TiO_2_-NPs), and gelatin (GL) using a solution casting technique, with and without varying volumes of garlic extract. The ability of fresh Nile tilapia fillets to retain their quality in the presence of various pollutants was examined (234). According to the findings, garlic extract-incorporated CMC/AG/GL/GE–TiO_2_ bio-nanocomposites demonstrated favorable thermal, mechanical, and morphological characteristics (234). The application of CMC/AG/GL/GE – TiO_2_ bio-nanocomposites containing 4% and 8% garlic extract on Nile tilapia fillets inhibited bacterial growth, reduced water loss during cold storage for 21 days (compared to uncoated samples), and extended the shelf life by one week (234).

By utilizing nano-coating technology, it is possible to extend the freshness of food significantly. To extend the shelf life of various products, antimicrobial and antioxidant properties are generated through the combination of hydrophilic and lipophilic substances, such as plant extracts or other carbohydrates, with metal nanoparticles, including zinc oxide, copper oxide, titanium dioxide, silver, and gold nanoparticles (235).

## 10 Conclusions

The potential of garlic in treating various chronic or acute conditions has been the subject of extensive research, as elaborated upon in the current review. The substantial abundance of phytochemicals present enhances many physiological processes. Protein, tryptophan, selenium, manganese, vitamins B1 and B6, and tryptophan are some nutrients found in garlic. The elements above synergize against a diverse range of health-threatening agents. However, further research is required to determine their exact mechanisms of action. Sulfoxide, s-methyl-1-cysteine, linalool, geraniol, and citral are a few of the sulfur-containing compounds found in garlic that have been associated with beneficial effects on health. Thiosulfinates and cysteine sulfoxides are two organic sulfur compounds associated with pharmacological activity. Garlic is increasingly recognized and utilized as fresh garlic juice, oil, garlic extract, and garlic powder, becoming increasingly popular as a natural remedy for various physiological disorders. Due to the unique amalgamation of bioactive constituents in *Allium* vegetables, their incorporation into our daily diet is imperative. Studies conducted *in vitro* and *in vivo* have demonstrated that garlic is beneficial in combating a range of metabolic disorders.

Garlic’s hypocholesterolemic, hypoglycemic, anticoagulant, antifungal, antioxidant, and antithrombotic attributes render it a multipurpose sustenance and medicinal substance. It is necessary to isolate and define garlic’s relative compounds and conduct additional research into its biological functions. Additional research is necessary to comprehend garlic’s mechanisms of action fully. Additional investigation is warranted to ascertain the potential impact of processing techniques such as fermentation and heat on garlic, as such knowledge could compromise the herb’s efficacy and safety. Further human clinical trials are required to validate the health benefits of garlic, with an emphasis on assessing its potential safety and adverse effects.

## Author contributions

ME-S: Writing – original draft, Formal analysis, Data curation. AS: Writing – original draft, Formal analysis, Data curation. SK: Writing – original draft, Software. HS: Writing – original draft, Resources. TAE-M: Writing – original draft, Software, Data curation. SA: Writing – original draft, Software, Formal analysis. ME: Writing – original draft, Software. SE: Writing – original draft, Software, Data curation. WM: Writing – original draft, Software, Resources. AAH: Writing – original draft, Software, Data curation. BM: Writing – original draft, Data curation. NA: Writing – original draft, Data curation. AAl: Writing – original draft, Software. ME-T: Writing – original draft, Software. SAQ: Writing – review & editing, Formal analysis. KE-T: Writing – review & editing, Investigation. SI: Writing – review & editing, Supervision, Resources.
